# Adaption of a dermal in vitro method to investigate the uptake of chemicals across amphibian skin

**DOI:** 10.1186/s12302-016-0080-y

**Published:** 2016-04-05

**Authors:** Katharina Kaufmann, Peter Dohmen

**Affiliations:** Department of Ecotoxicology, BASF SE, 67117 Limburgerhof, Germany

**Keywords:** Amphibians, *Xenopus laevis*, In vitro method, Skin permeability, Dermal absorption

## Abstract

**Background:**

Literature data indicate that terrestrial life stages of amphibians may be more sensitive to xenobiotics than birds or mammals. It is hypothesized that dermal exposure could potentially be a significant route of exposure for amphibians, as there is evidence that their skin is more permeable than the skin of other vertebrate species. Thus, higher amounts of xenobiotics might enter systemic circulation by dermal uptake resulting in adverse effects. Heretofore, no guidelines exist to investigate dermal toxicity of chemicals to amphibians. In order to minimize vertebrate testing, this work was targeted to develop an in vitro test system as a possible model to assess the dermal uptake of chemicals across amphibian skin.

**Results:**

The dermal absorption in vitro method (OECD guideline 428), an established toxicological (mammal) test procedure, was adapted to amphibian skin, in a first approach using the laboratory model organism *Xenopus laevis* and reference compounds (caffeine and testosterone). Skin permeability to both reference substances was significantly higher compared to published mammalian data. Caffeine permeated faster across the skin than testosterone, with ventral skin tending to be more permeable than dorsal skin. As usage of frozen mammalian skin is accepted, frozen skin of *X. laevis* was tested in parallel. To the freshly excised skin, however, freezing led to increased skin permeability, in particular to caffeine, indicating a loss of skin integrity due to freezing (without additional preservation measures).

**Conclusions:**

This work has demonstrated that the chosen method can be applied successfully to amphibian skin, providing the basis for further investigations. In future, well-established in vitro test systems and a broad dataset for many chemicals may help assess potential amphibian risk from xenobiotics without the need for extensive vertebrate testing.

**Electronic supplementary material:**

The online version of this article (doi:10.1186/s12302-016-0080-y) contains supplementary material, which is available to authorized users.

## Background

Amphibians are the vertebrate group most threatened by global decline. According to the IUCN Red List of threatened species 41 % of amphibian species were classified as being threatened in 2013. For birds and mammals, these percentages were estimated at 13.25 and 25 %, respectively [1]. Different reasons are discussed for this phenomenon. Four main causes are named for declining amphibian populations: loss of habitat (amphibians rely on both aquatic and terrestrial habitats), environmental pollution (including global climatic changes), diseases (fungal diseases in particular), and invasive species (predators and other pathogens) [2]. Amphibians may be exposed to various types of environmental contaminants such as various sources of unspecific anthropogenic pollution (waste water, deposits, air pollution), industrial or agricultural chemicals, or from traffic (directly or indirectly for example from salt used for de-icing) and climate change [3, 4]. Depending on their migration behavior and habitat selection, some amphibian species may be present on agricultural sites during times of fertilizer or pesticide applications and may thus be exposed to such chemicals [5, 6].

The current EU regulation 1107/2009 concerning registering plant protection products (PPP) requires assessing the risk to amphibians (and reptiles) from PPP. It had been assumed that the risk to amphibians is generally covered by studies with other vertebrate species (such as fish and birds/mammals). In fact, several data analyses show that available aquatic toxicity endpoints for fish and other aquatic organisms cover potential toxicity to aquatic life stages of amphibians [6, 7]. However, terrestrial life stages are not necessarily covered by studies of birds and mammals as indicated in recent literature findings [8, 9], which have demonstrated toxicity of several pesticides if sprayed directly on juvenile frogs. As toxicity data of birds and mammals mostly result from oral exposure scenarios, they are likely to cover the risk to amphibians from this route of exposure, too (Weltje et al. in review 2016). However, these data may not necessarily cover the risk to amphibians from dermal exposure to chemicals.

This work is based on the hypothesis that dermal exposure may be a relevant route for chemical uptake and toxicity in terrestrial life stages of amphibians, due to the particular properties and functions of amphibian skin. Among tetrapod vertebrates, the skin of amphibians is unique. Amphibian skin represents a compromise between maintaining a barrier to external conditions to protect the organism from desiccation and infections, and guaranteeing an intensive interaction with the environment to assure sufficient uptake of water, electrolytes, and oxygen. In contrast to the skin of terrestrial mammals, amphibian skin has only 1–2 cell layers of stratum corneum with no intercellular lipid layers [10]. The stratum corneum represents the outermost, keratinized part of the epidermis which is shown to be the main barrier for permeation of chemicals across mammalian skin [11, 12]. In addition, amphibian skin is highly glandular constituting another possible way for chemicals to penetrate the skin. Therefore, amphibian skin is supposed to be significantly more permeable as compared to other vertebrates, which might lead to higher amounts of xenobiotics entering systemic circulation by dermal absorption [4, 6, 13]. Thus, terrestrial life stages of amphibians may be more vulnerable from this route than other vertebrate species.

Thus far, dermal uptake of chemicals across amphibian skin is poorly understood and only few studies address amphibian skin permeability to xenobiotics, suggesting that amphibian skin is rather a weak barrier to the uptake of chemicals [14–16]. Currently, different physico-chemical properties are discussed to be possible predictors for the dermal uptake of a substance in amphibians. So far, little is known about influencing factors of chemical permeation across amphibian skin. Quaranta et al. showed frog skin permeability being linearly and positively correlated to log *P*_O/W_ (logarithm of octanol–water partition coefficient) of the applied substance, while log MW (logarithm of the molecular weight) did not predict dermal absorption [16]. Further, there is evidence from the literature that amphibian skin is permeable even to large molecules (>500 Da) and to a broader range of log *P*_O/W_ (−4 to +6) compared to mammalian skin [17]. Besides physico-chemical properties of a penetrant, the other important factor influencing dermal absorption is the skin structure itself, which may differ to some extent between species adapted to different habitats, but also within one animal between different skin sites such as dorsal skin, ventral skin, or the so-called pelvic patch as a skin area specialized for fast water uptake [18, 19].

Since there are currently no guidelines on dermal testing of amphibians, tests with amphibian skin may rely on existing toxicological test procedures for mammalian skin [14, 16]. Within human risk assessment, dermal in vitro methods are already established and routinely used to assess the dermal uptake of test compounds after topical application. As accepted by EFSA [20], the dermal absorption of test compounds may be investigated in vitro with excised mammalian—animal or human skin—or even reconstituted human skin models for the toxicological risk assessment of PPP in Europe. The skin is clamped between two chambers of a diffusion cell and the test substance is applied topically for a specific exposure period. At several time points, samples are taken out of the continuously stirred receptor medium beneath the skin and analyzed for the amount of test substance providing information on toxicokinetics. According to the guidelines for mammalian skin testing [12, 20, 21], both freshly excised skin and frozen skin are accepted for risk assessment as there is evidence that freezing has no significant effect on human skin permeability [22]. To assure that skin samples were not damaged by handling or storage, performance of an integrity test is required. In addition to the visual check of the skin sample for macroscopic damages, integrity may be tested by measurement of electrical resistance, transepidermal water loss, or the absorption of an internal standard across the skin [21]. Specific cut-off values derived from established historical data allow differentiation between damaged or intact skin samples.

The basic aim of this work was the adaption of a method to investigate the dermal uptake of chemicals in amphibians. Instead of animal testing, in vitro methods may contribute to reduce, refine, and replace (3 R’s) animal experiments. In order to minimize vertebrate testing, this work was targeted to adapt the dermal absorption in vitro method (OECD TG 428) as an established toxicological (mammal) test procedure to amphibian skin. Therefore, the skin of *Xenopus laevis* (African clawed frog) was used for these experiments. Although this is a species from African aquatic habitats and thus might not represent other more terrestrial amphibian species, it has decisive advantages for a first methodological development of the test system: *X. laevis* is worldwide established as a laboratory animal, is easy to maintain [23, 24], provides large pieces of skin due to its body size, and is not restricted to a special protection status (e.g., in Germany according to the Species Protection Act) in contrast to native species such as *Rana temporaria* or *Bufo bufo* [25], for which no specific laboratory cultures are available and which may need to be collected from the wild.

In order to compare the skin permeability of *X. laevis* to mammalian skin data, caffeine and testosterone were chosen as test compounds since they are well known as reference substances for performance with mammalian skin [12]. In addition, these two substances were chosen for their different physico-chemical properties, with caffeine being a model compound for a hydrophilic penetrant and testosterone for a lipophilic penetrant.

Three main questions were addressed in this study:

1. Is permeability dependent on the anatomical skin site? As mentioned above, structural properties of the skin might influence the dermal uptake of chemicals and skin structure may vary between different skin sites. Therefore, both dorsal and ventral skin samples were used to investigate potential differences.
2. Is it possible to use frozen skin as it is also validated for experiments with mammalian skin? The possibility to store skin samples would facilitate and shorten the complex experimental procedure. It would allow a more efficient usage of animals which would decrease the number of animals needed for experiments. Therefore, permeability of frozen stored skin was compared to permeability of freshly excised skin.
3. Is the impedance measurement a possible method to assure skin integrity prior to using a skin sample for an experiment as it is recommended by OECD guideline 428? Impedance measurement may be easily integrated into dermal absorption testing and would allow comparison to published amphibian skin impedance data. To this end, impedance data of the skin of *X. laevis* were collected in parallel to dermal absorption experiments.

## Results

### Dermal absorption of caffeine and testosterone

**Fig. 1:**
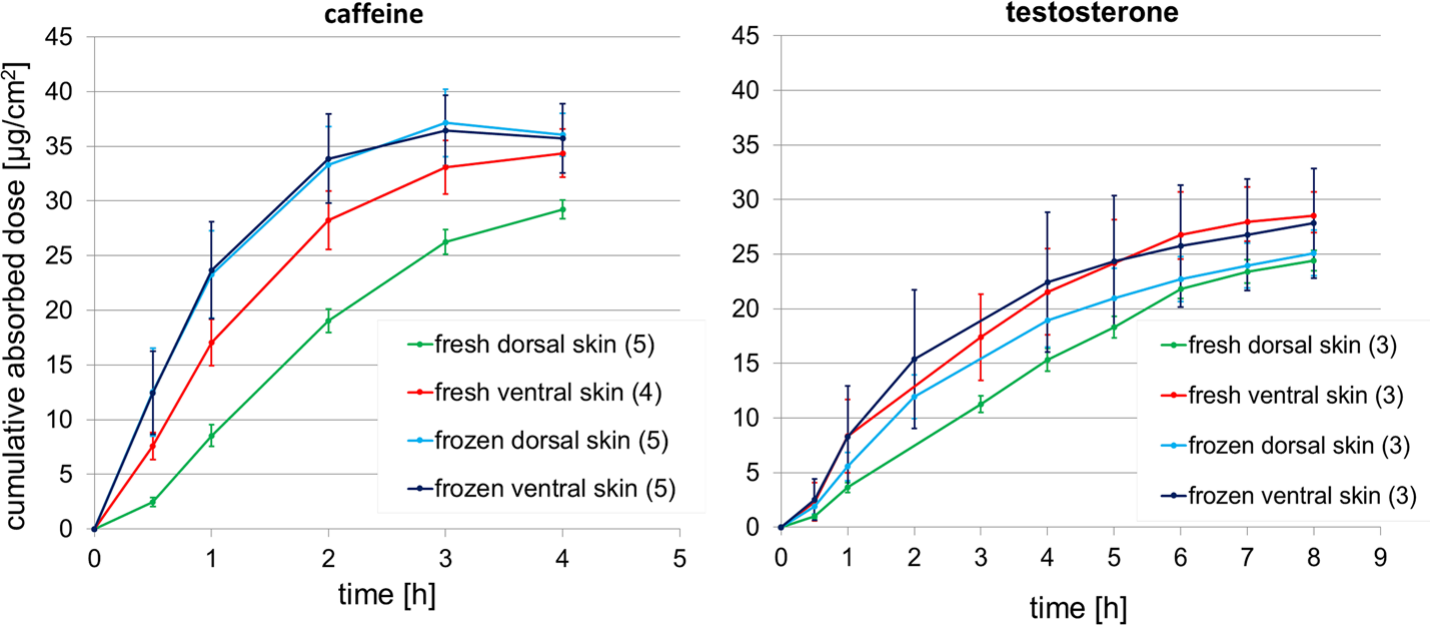
Absorption-time profiles of caffeine and testosterone permeation across the skin of *Xenopus laevis.* Mean cumulative absorbed dose ± standard deviation, found in the receptor fluids plotted against time and differentiated by skin storage and body side; mean values are based on 3–5 skin samples (as specified in *brackets* behind indication of skin storage and side in the diagram legends), stemming from one animal [except for testosterone data of freshly excised skin due to separation of testosterone results into 4- and 8-h exposure studies; see (Additional file 2) for 4-h testosterone data and detailed information on the individual cumulative absorbed doses]; due to incorrect sampling, testosterone data after 2 h for freshly excised skin and 3 h for frozen stored skin were excluded from this diagram

As shown by the absorption-time profiles, caffeine was taken up readily by the skin of *X. laevis* (Fig. 1). Within the first hour post-application, the steepest slope of caffeine permeation across freshly excised skin was reached. Maximum speed of caffeine absorption across frozen skin was achieved within the first half hour, indicating an increased permeability of frozen skin to this test compound. The mean cumulative dose absorbed by freshly excised dorsal skin did not increase as quickly as the other three absorption profiles by time, indicating that dorsal skin might be a greater barrier to permeation of caffeine than ventral skin. By calculation of mass balances at the end of exposure, the percentages of the applied dose of caffeine within the different diffusion cell compartments are shown (Table 1). Recoveries (sum of non-absorbed dose, skin content, and absorbed dose, only determined for radiolabeled studies) of both caffeine and testosterone were all within the acceptable range 100 ± 10 % [12]. Thus, data were regarded as valid and the application, skin washing, and extraction procedures were considered suitable to detect the chosen test compounds in the sample series of each diffusion cell.

**Table 1:** Mass balances of applied dose and kinetic parameters of caffeine and testosterone permeation across the skin of *Xenopus laevis*

Test compound	Caffeine				Testosterone			
Exposure time	4 h				8 h			
Skin	Fresh		Frozen		Fresh		Frozen	
	Dorsal (5)	Ventral (4)	Dorsal (5)	Ventral (5)	Dorsal (3)	Ventral (3)	Dorsal (3)	Ventral (3)
Non-absorbed dose [%]	5.7 ± 1.8	3.4 ± 0.3	1.4 ± 0.8	1.0 ± 0.9	12.9 ± 5.3	9.6 ± 1.8	21.5 ± 4.1	17.0 ± 10.1
Skin content [%]	20.6 ± 4.0 (2)	6.2 (1)	4.1 ± 1.2 (2)	–	16.5 ± 1.9	9.2 ± 5.0	9.5 ± 1.4	7.5 ± 3.5
Absorbed dose [%]	71.4 ± 2.2	87.5 ± 1.2	88.4 ± 5.0	90.3 ± 5.6	69.0 ± 3.3	84.8 ± 5.7	66.5 ± 6.3	75.2 ± 15.0
Recovery [%]	95.8 ± 2.0 (2)	97.9 (1)	98.1 ± 1.9 (2)	–	98.3 ± 3.6	103.6 ± 4.8	97.5 ± 2.6	99.8 ± 1.9
maxAR [µg/(cm^2^ × h)]	12.1 ± 1.2	19.0 ± 2.7	25.5 ± 7.2	26.8 ± 5.7	5.3 ± 0.5	12.0 ± 5.6	7.4 ± 1.3	11.4 ± 5.5
maxKp [×10^−3^ cm/h]	3.0 ± 0.3	4.7 ± 0.5	6.2 ± 1.5	6.7 ± 1.4	1.3 ± 0.1	3.0 ± 1.4	1.9 ± 0.3	2.9 ± 1.4

Mean values as percent of applied dose ± standard deviation based on n skin samples (*n* = 3–5, noted in brackets behind indication of skin site), stemming from two animals each [except for testosterone data of freshly excised skin due to separation of testosterone results into 4- and 8-h exposure studies; see (Additional file 1) for 4-h testosterone data and detailed information on the individual skin samples]

*non-absorbed dose* first and second skin washings and extraction of donor chamber, *skin content* amount recovered from the washed, digested skin, *absorbed dose* in receptor medium at exposure end and extraction of receptor chamber; recovery represents the sum of non-absorbed dose, skin content, and absorbed dose, *maxAR* maximum absorption rate, *maxKp* maximum permeability coefficient, calculated as described in “Methods” section

The highest percentages of caffeine were found in the receptor fluids, averaging 70 to 90 % of the applied dose [see (Additional file 1) for mass balances of each diffusion cell]. Only 1.0–5.7 % on average was washed off the skin surface at the end of exposure. Except for freshly excised dorsal skin, mean percentages of caffeine within the skin were rather low with 4.1–6.2 %, as well. Freshly excised dorsal skin showed a higher skin content of caffeine with 20.6 ± 4.0 %. Accordingly, the kinetic parameters: a maximum absorption rate of 12.1 ± 1.2 µg/(cm^2^ h) and a maximum permeability coefficient of 3.0 ± 0.3 × 10^−3^cm/h for freshly excised dorsal skin, which are less than half of the corresponding parameters for caffeine permeation across frozen stored skin.

The slope of the testosterone absorption curve is less steep indicating that the skin of *X. laevis* represents a greater barrier to the uptake of testosterone compared to caffeine. Nevertheless, the steepest slope was reached within the first hour of exposure (Fig. 1). Similar to caffeine experiments, testosterone permeated slowest across freshly excised dorsal skin. However, the differences between freshly excised and frozen skin and the location of the skin sites were not as clearly defined as for caffeine absorption [see (Additional file 2) for detailed information on cumulative absorbed doses for each time point]. Percentages of testosterone after 8 h found in the receptor fluids were slightly less than mean percentages of caffeine absorbed after 4 h, ranging between 66.5 ± 6.3 and 84.8 ± 5.7 % (Table 1). Non-absorbed doses of testosterone washed off the skin surface after 8 h ranged from 9.6 ± 1.8 to 21.5 ± 4.1 %. With regard to the content of testosterone within the skin, trends were similar to those of caffeine: the highest percentage of testosterone was contained within freshly excised dorsal skin accounting for 16.5 ± 1.9 % of the applied dose. The maximum absorption rates, ranging from 5.3 ± 0.5 to 12.0 ± 5.6 µg/(cm^2^ h), and maximum permeability coefficients, ranging from 1.3 ± 0.1 to 3.0 ± 1.4 × 10^−3^ cm/h, again demonstrated that the skin of *X. laevis* seemed to be a greater barrier for testosterone than caffeine uptake.

**Fig. 2:**
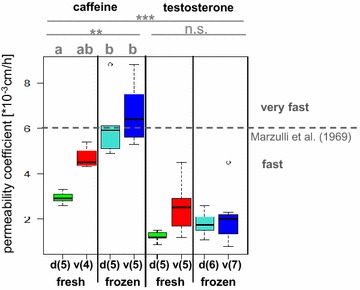
Comparative overview of permeability coefficients of caffeine and testosterone applied to the skin of *Xenopus laevis*. Permeability coefficients are arranged as boxplots, separated into the different groups fresh and frozen stored skin samples from dorsal (=d) and ventral (=v) body sides; each boxplot is based on 4–7 skin samples (as specified in *brackets* behind indication of skin side beneath the *boxplots*), stemming from two animals (for testosterone frozen stored skin samples stemmed from four frogs); *dashed* *gray line*: conferring to Marzulli et al. [26], substances may be classified into five classes for estimation of their permeation rates according to the obtained permeability coefficients (<6 × 10^−6^-, 6 × 10^−6^ to 6 × 10^−5^, 6 × 10^−5^ to 6 × 10^−4^, 6 × 10^−4^ to 6 × 10^−3^, and >6 × 10^−3^-cm/h meaning very slow, slow, moderate, fast, and very fast, respectively); *ns* not significant; *asterisks* indicate level of significance and different letters indicate significant difference calculated as described in “Methods” section. An Additional file shows individual maximum permeability coefficients in detail (see Additional file 1)

Maximum permeability coefficients which are independent of exposure duration were analyzed statistically as they represent comparable kinetic parameters (Fig. 2). The higher the permeability coefficient, the higher the permeability of the skin to the corresponding test substance. Describing the speed of a test substance permeation, certain ranges of permeability coefficients may be classified as ‘very slow,’ ‘slow,’ ‘moderate,’ ‘fast,’ and ‘very fast’ according to Marzulli et al. [26].

Permeability coefficients between caffeine and testosterone differed significantly (*p* < 0.001) when applied in vitro to the skin of *X. laevis*: the hydrophilic caffeine permeated faster across the skin than the lipophilic testosterone. However, according to Marzulli classes [26], both test compounds would be classified as being ‘fast’ permeating substances across freshly excised skin with a maximum permeability coefficient of less than 6 × 10^−3^ cm/h. For caffeine, significant differences (*p* < 0.01) could be found between the four tested groups (freshly excised dorsal and ventral and frozen dorsal and ventral skin), while for the same groups exposed to testosterone no statistically significant differences were observed (*p* = 0.14). However, similar trends were observed for testosterone: freezing tended to increase the permeability of the skin to testosterone and ventral skin seemed to be more permeable than dorsal skin. For caffeine, these trends were expressed more clearly: dorsal skin showed less permeability than ventral skin and frozen stored dorsal and ventral skin both showed a significantly increased permeability to caffeine compared to freshly excised dorsal skin (*p* < 0.05 and *p* < 0.01, respectively).

### Impedance measurements

In order to collect data to assess the suitability of impedance as a skin integrity parameter, the impedance of each skin sample was measured both at a frequency of 100 and 1000 Hz prior to each experiment. The most appropriate measurement frequency was found to be 100 Hz since measured impedances of the skin of *X. laevis* were low and impedance decreases with increasing frequency. The results shown refer only to measurements at 100 Hz. Further, impedance results were corrected by multiplying the measured impedance in Ω by the corresponding exposed skin area in cm^2^ as comparable impedances of amphibian skin within the literature are presented in the same way [27].

The impedance of frozen dorsal and ventral skin was 37 ± 254 and 263 ± 350 Ω cm^2^, respectively, and was significantly lower than the that of freshly excised dorsal and ventral skin with 779 ± 344 and 655 ± 406 Ω cm^2^, respectively (*p* < 0.001) (Additional file 3). Further, post hoc analysis revealed that no significant differences of the impedance may be found between dorsal and ventral skin samples of both freshly excised (*p* = 0.93) and frozen skin samples (*p* = 0.51). Thus, the anatomical origin of the skin samples had no significant effect on the impedance of the skin samples; however, standard deviations of all tested groups were considerably high. In line with the higher permeability of frozen skin to testosterone and in particular to caffeine (see above), some impedance data of frozen skin were measured within the negative range.

In addition to impedance data collection, two freshly excised, dorsal skin samples were damaged intentionally by perforating the clamped skin samples once with a lancet. Impedances for the two skin samples before perforation were 808 and 309 Ω cm^2^ and after perforation they decreased to 362 and 264 Ω cm^2^, respectively.

## Discussion

### General

The dermal absorption in vitro test system was successfully adapted to amphibian skin. The absorption of the two chosen test compounds caffeine and testosterone representing a hydrophilic and a lipophilic substance, respectively, differed significantly. Caffeine was shown to be absorbed considerably faster across the skin of *X. laevis* than testosterone. By trend, ventral skin seemed to be more permeable to both test compounds than dorsal skin, though especially to caffeine. Freezing of the skin enhanced the permeation of caffeine across the skin. For permeation of testosterone, there was a similar trend regarding differences between freshly excised and frozen stored skin; however, the differences were not statistically significant (at the low number of replicates and the observed variance). Comparative impedance measurements of amphibian skin revealed that impedance of freshly excised skin was significantly higher than that of frozen stored skin, which included negative values in single cases. However, impedance data were rather low and showed high variability, indicating that this method may not be sufficient at this stage to check skin integrity until further investigations.

### Comparison to mammal skin permeability

In comparison to mammal skin data, the skin of *X. laevis* was shown to be considerably more permeable to caffeine and testosterone. On average, 71.4 % (dorsal) to 87.5 % (ventral) of the applied dose of caffeine was found in the receptor fluid after 4 h. In an inter-laboratory data collection of human skin in vitro, mean measured percentages in receptor fluids ranged from 10.9 to 46.5 % of the applied dose after 24 h [28]. Although exposure time was 6 times higher, clearly less was absorbed through human skin compared to the skin of *X. laevis*. For testosterone, it was shown that on average 69.0 % (dorsal) to 84.8 % (ventral) of the applied dose was permeated across the skin of *X. laevis* after 8 h. The corresponding data shown by van de Sandt et al. [28] again demonstrate that after 24 h only 3.9–38.9 % of the applied dose of testosterone is permeated across human skin on average. For pig skin (dermatomed skin, 500–900 µm thick), data were found from static diffusion cell experiments, measured under conditions which are comparable to those of this study (the applied dose of caffeine and testosterone was 40 µg/cm^2^, test compound vehicles were water for caffeine and ethanol for testosterone, and the receptor fluid was composed of a Ringer’s solution including 4.5 % BSA for testosterone) [29]. Mean permeability coefficients of pig skin derived from this study were 0.25 × 10^−4^cm/h and 0.20 × 10^−4^cm/h for caffeine and testosterone, respectively. Average maximum permeability coefficients of amphibian dorsal and ventral skin measured in our study were 37 × 10^−4^cm/h and 19 × 10^−4^cm/h for caffeine and testosterone, respectively. Thus, compared to pig skin, permeability of amphibian skin (*X. laevis*) is 148- and 95-fold greater to caffeine and testosterone, respectively. The magnitude of these differences between amphibian and pig skin corresponds to the results reported by Quaranta et al. [16], who directly compared amphibian (*Rana esculenta*) and pig skin by a similar method in vitro. Compared to pig skin permeability, they found amphibian skin permeability being 26-, 29-, 66-, 120-, and 302-fold increased to glyphosate, paraquat, mannitol, antipyrine, and atrazine, respectively.

The higher permeability of amphibian skin may be attributed to structural differences within the skin. The skin of terrestrial mammals such as the skin of pigs or humans is characterized by a multi-layered stratum corneum, which is also supposed to be a main barrier to dermal uptake of xenobiotics [11]. The stratum corneum of amphibians is only about 2 µm thick and consists of only 1–2 cell layers [30], while that of humans may be composed of 15–30 cell layers with a thickness of about 10–20 µm, depending on the body region [31]. The stratum corneum of terrestrial mammals is not only considerably thicker than that of amphibians, it is also characterized by lipids secreted by cellular lamellar granules (such organelles are not present in amphibian skin) and exocytosed into the extracellular matrix. Therefore, intercellular spaces are completely filled with these lipids arranged in multiple sheets building a continuous compartment within the stratum corneum [10, 32].

As mentioned above, the differences between mammalian and amphibian skin are large; this is particularly true for the less lipophilic compound (see above). This fact may be attributed to the thinner stratum corneum and particularly due to the lack of intercellular lipids in amphibians.

Hypothetically, molecules may be transferred across the stratum corneum by three different pathways [33]: (1) paracellular (the substances diffuse through intercellular lipids), (2) transcellular (through the cellular compartment), and (3) transappendageal (substances circumvent keratinocytes by hair shunts or glands). For mammalian skin, the paracellular pathway is believed to be the major transfer route where substances cross the lipid-rich regions in between the corneocytes of the multi-layered, non-viable stratum corneum. The transcellular and transappendageal pathways are considered as being less likely for the transfer across the stratum corneum [33–35]. For amphibians, the transcellular pathway is hypothesized to be more relevant than the paracellular route since the outermost cells of the stratum corneum and granulosum are connected by tight junctional structures [30, 36]. Apart from stratum corneum differences, glandular tissue is far more pronounced in amphibian skin than in mammalian [10]. Thus, transappendageal routes might play a significant role for dermal absorption of chemicals in amphibians. Via glandular ducts of granular and mucous glands, chemicals might circumvent stratum corneum and viable epidermal layers and might be transported directly into the dermis, reaching blood vessels faster for resorption. In mammalian skin, an increased amount of appendages such as hair shafts may lead to an increased absorption of chemicals, as well [37, 38].

Although amphibian skin is widely assumed to be more permeable than mammalian skin due to the special structure and functions, quantitative comparisons are rare. The results of this work further support the hypothesis that amphibian skin is more permeable to xenobiotics than mammalian skin and represents a weaker barrier to the dermal uptake of chemicals.

### Dermal absorption of caffeine and testosterone

Freshly excised skin of *X. laevis* absorbed between 71.4 % (dorsal) and 87.5 % (ventral) of the applied dose of caffeine on average 4 h post-application. For testosterone, mean absorbed percentages were similarly high after 8 h (69.0 % across dorsal to 84.8 % across ventral skin). So far, no information on the dermal absorption of these reference compounds across amphibian skin may be found in the literature. However, there is evidence that corticosterone, a steroid hormone such as testosterone, may be absorbed by the dorsal skin of different species of terrestrial salamanders and frogs, leading to a fivefold increase in blood levels in vivo [39]. With regard to the magnitude of caffeine and testosterone percentages absorbed across amphibian skin, the results of this work correspond to absorbed percentages of other test compounds such as malathion, parathion, or carbaryl ranging from 76.8 to 96.2 % after 6–8 h post-application [14, 15], indicating that amphibian skin is a rather weak barrier to xenobiotics.

Although high percentages of both tested compounds were taken up by the skin of *X. laevis*, caffeine permeation was significantly faster than testosterone permeation. This difference may be attributed to the differing physico-chemical properties of the two substances. Modeling dermal absorption of test compounds by physico-chemical properties may be approached by the so-called quantitative structure–permeability relationships (QSPR). Main determinants of dermal absorption are indicated to be molecular size (expressed as molecular volume or weight) and hydrophobicity (expressed by log *P*_O/W_). Basically, in mammalian skin low molecular weight and high lipophilicity (up to a log *P*_O/W_ of 3) lead to higher permeability coefficients [17, 40]. In contrast to mammalian skin, the skin of *X. laevis* is characterized by a superficial mucus film. Thus, dermal penetration of lipophilic compounds such as testosterone (log *P*_O/W_ = 3.32 [28]) might be impeded, while caffeine (log *P*_O/W_ = 0.01 [28]) is likely to partition faster from the applied dose solution into the aqueous mucous layer and viable epidermis. Besides lipophilicity, also the higher molecular weight of testosterone compared to caffeine might lead to a retarded penetration into amphibian skin.

Results of this work do not correspond to those of Quaranta et al. [16], who demonstrated that substances with higher log *P*_O/W_ permeated faster across frog (*Rana esculenta*) skin than substances with a lower log *P*_O/W_ in vitro. Van Meter et al. [41] exposed different amphibian species to different pesticides (imidacloprid, atrazine, triadimefon, fipronil, and pendimethalin) via treated soil in vivo and measured amphibian body burdens. They observed that log *P*_O/W_ was not related to body burden, while water solubility or soil partition coefficients were better estimators for this endpoint. At this stage, no clear conclusions on the relationship between physico-chemical parameters and amphibian skin permeability can be drawn and more data are needed to identify the influence of these parameters.

### Differences between dorsal and ventral skin

By trend, freshly excised ventral skin of *X. laevis* was more permeable to both test compounds than dorsal skin with mean maximum permeability coefficients of 4.7 × 10^−3^cm/h for caffeine and 3.0 × 10^−3^cm/h for testosterone and 3.0 × 10^−3^cm/h for caffeine and 1.3 × 10^−3^cm/h for testosterone, respectively. One reason for increased permeability of ventral skin might be its decreased thickness. Dorsal skin of *X. laevis* was measured to be 100 µm thicker (622 ± 108 µm) than ventral skin (523 ± 83 µm), and maximum permeability coefficients were found to be negatively correlated to skin thickness (statistical analysis restricted to freshly excised skin; caffeine: Pearson’s product-moment correlation, moderate correlation, *r* = −0.65*; testosterone: Spearman’s rank correlation rho, strong correlation, *r* = −0.84**). Decreased skin thickness in amphibian skin usually goes along with a decreased amount of epidermal cell layers which would imply a decreased passage length for diffusing test compounds across this skin layer [17]. However, further morphological characteristics such as the amount of glands within the skin might influence dermal absorption of test compounds. So far, only few studies addressed permeability differences of dorsal and ventral skin sites in amphibians. As *X. laevis* is an aquatic amphibian species, it is not characterized by ventral skin areas specialized for water uptake [42]. Thus, considerable permeability differences were not necessarily expected for this species. However, results of this work correspond to those of Willens et al. [14], who measured the average permeability coefficient of dorsal skin of *Rana catesbeiana* exposed to malathion in vitro being lower (6.5 × 10^−3^cm/h) than that of ventral skin (8.95 × 10^−3^cm/h). These authors argue that thickness of the outermost stratum corneum might explain observed differences as mean measured dorsal stratum corneum was thicker than ventral stratum corneum on average. As the stratum corneum plays a decisive role as a barrier to the uptake of substances in mammalian skin, this is likely to be a relevant factor in amphibian skin as well. Further, Willens et al. demonstrated that glandular tissue was more abundant in dorsal than in ventral frog skin and hypothesized that glandular tissue may represent a barrier to absorption by secretion of additional absorption barriers and creation of a depot effect which would explain higher percentages within dorsal skin samples and retarded uptake of malathion across this skin side. However, a detailed comparative histological investigation of dorsal and ventral skin structures of *X. laevis* is still lacking.

Another difference between dorsal and ventral skin found in this work was that mean skin contents of caffeine at the end of exposure tended to be higher in dorsal (20.6 %) than ventral skin (6.2 %). For testosterone, similar differences between dorsal and ventral skin contents were found. In female *X. laevis*, the area of skin glands is increased in dorsal skin compared to ventral skin [43]. An increased area of glandular tissue in dorsal skin may lead to an increased accumulation of test compounds, which permeated across the skin via the transappendageal pathway. As mentioned above, similar trends were observed by Willens et al. [14].

Dermal exposure of terrestrial life stages of amphibians to xenobiotics such as pesticides may take place by direct overspray or uptake from treated surfaces such as soil or plants. Therefore, both dorsal and ventral skin areas might come into contact with xenobiotics. Given the fact that some amphibian species actively absorb water from moist surfaces by their specialized pelvic patch, such skin areas might further increase uptake of xenobiotics. Apart from skin specializations of amphibian species which are adapted to terrestrial habitats, structural differences between dorsal and ventral skin such as skin thickness or the abundance of glands might lead to different permeability characteristics as shown here for *X. laevis*. Further studies will be needed to conclude whether skin thickness is a main parameter responsible for permeability differences or whether different skin structures between different parts of the body and furthermore between species drives the observed permeability differences.

### Differences between freshly excised and frozen stored skin

Storing skin samples in aluminum foil directly after excision and placing them at −24 ± 2 °C without any additives is a common method for mammalian skin samples [44, 45]. As shown in this work, skin permeability of *X. laevis* in particular to caffeine was significantly increased by this freezing method. The reason for increased permeability of frozen stored skin of amphibians in contrast to frozen stored skin of mammals might be again due to structural differences. In mammalian skin, the lipid-rich stratum corneum as a main barrier for the penetration of chemicals is not affected considerably in its barrier function by freezing [22]. In contrast, the skin of *X. laevis* is highly glandular and covered by mucus and therefore characterized predominantly by a hydrous matrix. Therefore, amphibian skin might be more vulnerable to ice crystal formation due to freezing. The fact that caffeine absorption is apparently more severely increased in frozen stored skin than testosterone suggests differential absorption pathways of the two test compounds. In contrast to the lipophilic testosterone, caffeine might be absorbed predominantly via hydrophilic, transcellular, or transappendageal pathways including more hydrous matrices which might be affected considerably by ice crystal formation-caused disruptions.

The results of this work indicate that freezing of the skin without any preservation measures is unsuitable for dermal absorption in vitro studies with amphibian skin. Nevertheless, the possibility to work with stored skin samples would be very useful as it would lead to a higher flexibility and reduced time consumption on the day of the actual experimentation and it would allow a more efficient usage of animals since the amount of skin samples per experiment is limited (animal welfare reasons). Thus, further storage methods should be investigated, for example by adding anti-freezing agents to the skin samples in order to minimize ice crystal formation.

### Impedance measurement as a future skin integrity test

The impedance of freshly excised skin measured at a frequency of 100 Hz was found to be in the range of 779 ± 344 Ω cm^2^ and 655 ± 406 Ω cm^2^ (dorsal and ventral, respectively, correlating to the respective lower ventral skin thickness). These values for the skin of *X. laevis* correspond to impedance data shown by Watkins and Dennis [27], who measured impedance of freshly excised ventral skin of *Rana pipiens* ranging from 236 to 1400 Ω cm^2^ at a frequency of 50 Hz. This slightly lower measurement frequency might explain the slightly higher impedances, ranging up to 1400 Ω cm^2^.

In line with the observed permeability differences, impedances of frozen skin samples were significantly lower than those of freshly excised skin samples indicating that freezing of the skin might affect impedance, possibly by affecting skin integrity. From this point of view, it might be suggested that impedance measurement is suitable for the assessment of skin integrity. In theory, epithelia such as amphibian skin are characterized by tight junctions, which separate apical from basolateral membranes. The tightness of adjacent cells sealed by these tight junctions determines the integrity of an epithelium [46]. By freezing, possible ice crystal formation followed by bursting of some epidermal cells might lead to impairment of this tight barrier, and consequently impedance decreases.

Further, single frozen skin samples were shown to exhibit negative impedances. Since existence of negative skin impedances is unlikely, such individual cases might be rather attributed to inaccurate fixing of the electrode in the donor chamber before or after clamping the skin into the diffusion cells or other unknown influencing parameters. Even slight differences in the distance from the tip of the donor electrode to the skin surface may lead to considerably different impedance results, indicating the susceptibility of impedance measurement for slight methodical inaccuracies or external influences. Furthermore, measured impedance ranges of freshly excised and frozen skin of *X. laevis* overlapped clearly and impedances of freshly excised skin of *X. laevis* were rather low. Intact human skin samples are usually characterized by an impedance of higher than 1 kΩ at 1 kHz and may be rejected below this range based on historical datasets and depending on laboratory-specific conditions [47]. Due to these low impedances of amphibian skin, only little room would be left for the detection of impaired skin samples and transition between intact and impaired skin might be smooth and overlapping. This was also shown by the additional perforation test of two dorsal skin samples, which decreased only about 15 and 50 % in their impedances relative to their original impedances before perforation. Accordingly, when impedance is already low, a structural damage such as a hole in the skin sample is difficult to detect. However, since the check of skin integrity is essential for the permeability test, impedance measurement does not appear to be suitable for this purpose and the derivation of an impedance cut-off value to distinguish impaired from intact skin samples might be difficult. Additional investigation into other skin integrity tests should be evaluated for suitability, such as the measurement of transevaporative water loss or the absorption of internal standards (e.g., methylene blue). However, as integrity tests are based on species-specific historical datasets, a broad collection of data is necessary for the derivation of cut-off values.

## Conclusions

Terrestrial life stages of amphibians may be more sensitive to chemicals than other vertebrate species due to the particular properties and functions of their skin. In accordance with limited literature data, these studies have demonstrated that amphibian skin may be significantly more permeable to chemicals than mammalian skin. Increased permeability of amphibian skin compared to mammalian may be attributed to biochemical and structural skin differences. Thus, dermal exposure to environmental contaminants can be a relevant route of exposure for amphibians. So far, no guidelines exist to investigate dermal uptake of chemicals across amphibian skin. The present work has shown that the dermal absorption in vitro method according to OECD guideline 428 may be successfully adapted to the skin of the amphibian *X. laevis*, providing the basis for further investigations. However, further investigations are needed to assess whether *X. laevis* can be used as a representative model system for other species, too. Prospective work should evaluate species differences including morphological investigations of different skin sites in parallel to dermal absorption studies. Besides structural aspects of the skin, physico-chemical properties should be examined as factors influencing dermal absorption by collecting comparable permeability data of different types of chemicals. Such an established in vitro method and a broad dataset for many chemicals may help assess potential amphibian risk from xenobiotics without the need for extensive animal testing.

## Methods

### Animal husbandry

Adult African clawed frogs (*X. laevis*, wild type) with a length of 10–11 cm (measured from snout to vent) and a weight varying from 111 to 173 g were obtained from Xenopus Express France (Vernassal, Haute-Loire, France) and held in a laboratory at BASF SE, Limburgerhof, Germany. Female frogs were chosen due to their larger body size allowing excision of large skin areas in order to minimize the numbers of animals needed (animal welfare reasons).

The frogs were kept in shaded tanks with PVC tubes serving as a shelter and maintained at a temperature of 20 ± 1 °C. They were fed with fish food pellets, beef liver, sludge worms (*Tubifex tubifex*), or harlequin fly larvae (*Chironomus riparius*) three times a week. After feeding, the water was completely changed and water quality was measured regularly. The animals were allowed to acclimatize in the laboratory for 5 weeks before starting the experiments. In total, the dorsal and ventral skin of seven frogs was prepared successively for the dermal absorption experiments. Husbandry and euthanasia conditions complied with the directive 2010/63/EU of the European Parliament and of the Council on the protection of animals used for scientific purposes [48].

### Preparation of skin samples

For euthanasia, the frogs were immersed in a neutral (sodium bicarbonate-buffered) 0.4 % MS222 (ethyl 3-aminobenzoate methanesulfonate, Sigma-Aldrich Chemie GmbH, Steinheim, Germany) for 15 min. As recommended by the American Veterinary Medical Association [49], death was assured by decapitation and destruction of the spinal marrow. Euthanized frogs were rinsed in tap water to wash off superficial residues of MS222. Full-thickness (confirmed by histological observations: epidermis, dermis, and hypodermis), circular skin samples with a diameter of 2.5 cm from the dorsal and ventral skin area were prepared by excising the dorsal and ventral skin in one piece and punching out the samples on a cutting board. During the preparation process, the skin was kept moist with amphibian Ringer’s solution (prepared freshly for each dermal absorption experiment according to Gentz [50]: 112.9 mM sodium chloride, 1.3 mM potassium chloride, 2.0 mM calcium chloride, and 2.4 mM sodium bicarbonate. Four skin samples were obtained from each area (dorsal and ventral), located bilaterally in two rows along the midline from cranial to caudal. Typically, six freshly excised skin samples were run in parallel, and the remaining skin samples of a frog were stored in aluminum foil at −24 ± 2 °C (at minimum for 24 h) until further use.

### Chemicals and dose solutions

Non-radiolabeled compounds, caffeine (3,7-dihydro-1,3,7-trimethyl-1H-purine-2,6-dione, CAS no. 58-08-2) and testosterone (4-androsten-17β-ol-3-one, CAS no. 58-22-0), were purchased from Sigma-Aldrich Chemie GmbH, Steinheim, Germany. Radiolabeled compounds ([1-methyl-^14^C] caffeine and [4-^14^C] testosterone) with a specific radioactivity of 2.0 GBq/mmol and 2.2 GBq/mmol, respectively, were provided by American Radiolabeled Chemicals, Saint Louis, MO, USA. Due to comparability, the dose solutions of each compound were prepared at a target concentration of 4 mg/mL since this is usually the applied concentration in reference studies on mammalian skin [28]. Caffeine dose solutions were prepared in amphibian Ringer’s solution, while testosterone was dissolved in ethanol/water 1/1 (v/v). For radiolabeled studies, non-radiolabeled and radiolabeled compounds were mixed, yielding the target concentration of 4 mg/mL at a radioactivity in the range of 1–1.5 MBq/mL. Test substance concentration or radioactivity of every dose solution was verified after preparation, before and directly after application to the skin samples. The actual applied concentrations were calculated based on the mean measured concentration directly before and after application.

### Dermal absorption studies

The dermal absorption in vitro studies with amphibian skin were conducted according to OECD guideline 428 [12] with some adjustments necessary for the adaption to amphibian skin. The experiments were run at room temperature (21 ± 1 °C). After measuring skin thickness, the skin samples were mounted on static Franz diffusion cells with an average exposed skin area of 1.85 cm^2^ and a receptor volume of 12.5 mL [BASF SE, Ludwigshafen, Germany (see Additional file 4 for schematic overview of a diffusion cell)]. For detailed information on the properties of each skin sample, refer to Additional file 3. Frozen skin samples were allowed to thaw at room temperature (21 ± 1 °C) for a minimum of 30 min in amphibian Ringer’s solution. For caffeine studies, the receptor medium consisted of acclimatized amphibian Ringer’s solution. For testosterone studies, 5 % bovine serum albumin [BSA, Albumin Fraction V (pH 7.0) Blotting grade, AppliChem GmbH, Darmstadt, Germany] was added to freshly prepared amphibian Ringer’s solution as it is an approved method in reference studies, enabling the transfer of testosterone to the receptor medium [21, 28]. The diffusion cells were placed on multiple-site magnetic stirrers (RO 5 power, IKA-Werke GmbH and Co. KG, Staufen, Germany) to keep the receptor medium homogenous throughout the sampling period (magnetic cross stir bars, 5 × 10 mm, VWR International GmbH, Darmstadt, Germany).

In order to test if electrical resistance measurement is suitable for skin integrity assessment, impedance (resistance to an alternating current) data were collected as follows: the donor chamber was filled up with amphibian Ringer’s solution and the electrodes of an LCR meter (*L* = inductance, *C* = capacitance, *R* = resistance; LCR Meter ST2822, Sourcetronic GmbH, Bremen, Germany) were placed into the electrolyte solution within receptor and donor chamber, fixed in their position to maintain the distance to the skin surface throughout the experiment. Impedances were measured both at a frequency of 100 and 1000 Ω for each skin sample. For calculating the impedance value of the skin itself, the inherent impedance of each chamber, filled with the corresponding receptor medium, was subtracted from the measured impedances when the skin samples were clamped into the diffusion cells. The receptor medium within each donor chamber was removed and the surface of the skin was gently wiped with a cotton swab in a standardized way to remove the remaining medium.

A volume of 10 µL/cm^2^ of the prepared dose solutions was applied topically to the skin, representing a finite dose of 40 µg/cm^2^. After 0.5, 1, 2, 3, and 4 h of exposure (for one part of the testosterone studies exposure time was extended to 8 h), aliquots of the receptor medium were collected and replaced by fresh receptor medium with syringes (disposable needles, Sterican, 0.80 × 80 mm, 21 G × 3 1/8″, B. Braun Melsungen AG, Melsungen, Germany, connected to disposable syringes, Norm-Ject, 2 mL, Luer-Lock, Henke-Sass Wolf GmbH, Tuttlingen, Germany). Between the time points of aliquot collection, the inlet to the receptor chamber was covered by a piece of Parafilm M^®^ laboratory film (Bemis Company, Inc., Oshkosh, WI, USA) to prevent evaporation of the receptor medium. The donor chamber was covered by a piece of Fixomull^®^ (BSN medical GmbH, Hannover, Germany) moistened regularly with amphibian Ringer’s solution to avoid desiccation of the skin.

At the last sampling time point, the receptor medium was drawn out of the receptor chamber completely. Each diffusion cell was dismantled to analyze the amount of test compound in the different compartments: to determine the remained amount of test compound on the skin surface, the skin was washed by alternately pipetting 1 mL of the corresponding solvent of the test substance up and down on the skin surface and wiping the skin with cotton swabs dampened into the solvent. This procedure was repeated two times, followed by wiping the skin surface with one dry cotton swab. All cotton swabs, pipettes, and pipetted solvents of one diffusion cell were collected in one vessel and extracted in the corresponding extraction fluid (water for the extraction of caffeine and 100 % ethanol for the extraction of testosterone). This step represented the first skin wash. In the next step, the donor chamber was removed from the skin surface and wiped at the bottom with a cotton swab soaked in solvent, followed by a dry cotton swab. The donor chambers were extracted with the cotton swabs and the covering piece of Fixomull^®^ in a second series of vessels. The skin was wiped by two cotton swabs (one soaked in solvent and one dry) a second time for extraction. In order to determine the amount of test compound within the skin in case of radiolabeled studies, the skin was removed from the receptor chamber and digested in 8 mL of a tissue solubilizer (Soluene^®^ 350, PerkinElmer Health Sciences B.V., Groningen, the Netherlands). For determination of the amount of test compound in the receptor medium in addition to aliquots at the end of exposure, the receptor chambers were extracted with the covering piece of Parafilm M^®^ on the inlet and the magnetic stir bars in the corresponding extraction fluid. Volumes of extraction fluids and aliquots of receptor media at various time points were calculated by subtraction of net weights from filled weights of all vials.

### Analytics

For both test compounds, radiolabeled studies were conducted in order to check if recoveries are within an acceptable range (100 ± 10 % according to OECD guideline 428). Experiments with caffeine were conducted preferably non-radiolabeled. However, testosterone experiments were conducted exclusively radiolabeled as the receptor medium in this case contained protein (5 % BSA) which would interfere with HPLC–UV detection. All samples of an experiment were homogenized directly before taking aliquots for analysis.

Non-radiolabeled caffeine samples were analyzed by HPLC–UV detection (high-performance liquid chromatography; pump: 2695, detector: 2996 PDA (Alliance HPLC system), Waters Corporation, Milford, MA, USA; column: Synergi Hydro-RP, 80 A, 4 µ, 150 × 3 mm, Phenomenex Inc., Aschaffenburg, Germany; injection volume: 50 µL; flow rate: 0.7 mL/min.; eluent: 85 % formic acid in deionized water (1 mL/L) and 15 % formic acid in acetonitrile (1 mL/L); UV detection wavelength: 273 nm). Concentrations of caffeine in the samples were calculated by relating the measured peak areas [analyzed via corresponding software ‘Chromeleon 6.80 SR9′ (© 1994–2010 Dionex Corporation)] to those of known caffeine concentrations freshly prepared for each run for calibration purpose. Concentrations below the calibration line range (<40 µg/L) were considered as containing no caffeine.

Radiolabeled samples were mixed with liquid scintillation cocktails (Irgasafe Plus or Hionic Fluor, PerkinElmer Health Sciences B.V., Groningen, the Netherlands) and analyzed for total radioactivity by LSC (liquid scintillation counting; Wallac 1414 or Tri-Carb^®^ 2910TR, PerkinElmer, Inc., Waltham, MA, USA).

### Data evaluation

In the present work, the sum of test substance contained in first and second skin washings and donor chamber extraction was regarded as the ‘non-absorbed dose.’ The ‘absorbed dose’ was considered to be the amount of test substance within the receptor fluid at exposure termination and the extraction of the receptor chamber. The mass of test substance found in digested skin samples contributed to the ‘skin content.’

Data of HPLC and LSC analyses were processed in an Excel sheet for calculation of mass balances of each diffusion chamber expressed in per cent of the applied dose (recoveries only quantifiable for radiolabeled studies) and absorption-time profiles (cumulative absorbed dose in µg/cm^2^ plotted against time, Fig. 1). On the basis of the absorption-time profiles, kinetic parameters were calculated [the steepest slope between two time points represented the maximum absorption rate (maxAR) in µg/(cm^2^ h] and division of maxAR by the applied concentration of the test substance provided the maximum permeability coefficient (maxKp) in cm/h).

**Fig. 3:**
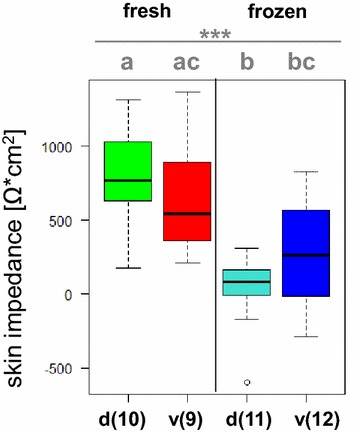
Comparative overview of impedances of the skin of *Xenopus laevis* prior to dermal absorption experiments. Measured skin impedance data illustrated as boxplots separated into freshly excised and frozen stored skin with dorsal (=d) and ventral (=v) body sides; each boxplot is based on 9–12 skin samples (as specified in *brackets* behind indication of skin side beneath the boxplots), stemming from four (freshly excised skin) to six (frozen stored skin) animals each; *asterisks* indicate the level of significance and different letters indicate significant difference calculated as described in “Methods” section. An Additional file shows individual measured impedances in detail (see Additional file 3)

Statistical analyses of dermal absorption data were performed with R version 3.0.1 (The R Foundation for Statistical Computing 2013). Maximum permeability coefficients of this work are shown by a boxplot diagram (Fig. 2). Comparison between caffeine and testosterone data is based on a ‘Wilcoxon rank sum test with continuity correction.’ One-way analysis of variance within caffeine data was performed with a ‘Kruskal–Wallis rank sum test’ followed by a post hoc ‘Pairwise comparisons using Tukey and Kramer (Nemenyi) test.’ One-way analysis of variance within testosterone data was performed with a ‘Kruskal–Wallis rank sum test.’ Impedance data are shown by a boxplot diagram, as well (Fig. 3). One-way analysis of variance of impedance measurements between the different groups was performed by a ‘Kruskal–Wallis rank sum test’ followed by a post hoc ‘Pairwise comparisons using Tukey and Kramer (Nemenyi) test.’ Boxplots with the same letters do not differ significantly. Statistically significant (*p* value <0.05), very significant (*p* value <0.01), and highly significant (*p* value <0.001) results are denoted by asterisks ‘*,’ ‘**,’ and ‘***,’ respectively.

## Additional files

10.1186/s12302-016-0080-yPercentages of the applied dose and kinetic parameters of dermal absorption in vitro experiments. Skin samples are listed according to treatment (test compound and storage); Xlf = *Xenopus laevis*, female; used animals are numbered consecutively (2–7); skin side is described as d = dorsal or v = ventral, first number describes area of skin side (1 = cranial, 2 = caudal) and second number describes if the sample stemmed from the left (= .1) or the right side (= .2) related to the top view of the animal; non-absorbed dose = first and second skin washings + donor chamber extraction, skin = amount recovered from the washed, digested skin; absorbed dose = in receptor medium at exposure end and extraction of receptor chamber; recovery represents the sum of non-absorbed dose, skin content and absorbed dose; maxAR = maximum absorption rate, maxKp = maximum permeability coefficient, calculated as described in Methods; nd = not determined.
10.1186/s12302-016-0080-yCumulative absorbed doses of dermal absorption in vitro experiments. Each skin sample represents one diffusion cell and is listed according to treatment; cumulative absorbed doses found in the receptor fluids of each diffusion cell are listed for each sampling time point; Xlf = *Xenopus laevis*, female; used animals are numbered consecutively (2-7); skin side is described as d = dorsal or v = ventral, first number describes area of skin side (1 = cranial, 2 = caudal) and second number describes if the sample stemmed from the left (= .1) or the right side (= .2) related to the top view of the animal.
10.1186/s12302-016-0080-yCharacteristics of skin samples used for dermal absorption in vitro experiments. Skin samples are listed according to treatment (test compound and storage); *Xlf* = *Xenopus laevis*, female; used animals are numbered consecutively (2–7); skin side is described as d = dorsal or v = ventral, first number describes area of skin side (1 = cranial, 2 = caudal) and second number describes if the sample stemmed from the left (= .1) or the right side (= .2) related to the top view of the animal; prior to experiments, skin thickness and impedance (multiplied by the skin area) of each skin sample was measured as outlined in “Methods” section.
10.1186/s12302-016-0080-ySchematic overview of a diffusion cell. Skin samples are clamped in between the receptor and donor chamber and fixed by a metal clamp (for clarity, the metal clamp is not shown in this Figure); the receptor chamber is filled with the corresponding receptor medium which can be temperature-controlled via an outer chamber; test compounds are applied topically to the skin in the donor chamber and samples are drawn out of the receptor medium across the connection pipe of the receptor chamber; at the end of exposure remaining test substance may be washed off the skin surface and the diffusion cell may be dismantled; diffusion cell parts can be extracted separately for mass balance and all samples are analyzed.
