# Supplementary material for: Adaption of a dermal in vitro method to investigate the uptake of chemicals across amphibian skin
Source: Environ Sci Eur. 2016 Apr 5;28(1):10. doi: 10.1186/s12302-016-0080-y (PMC5044961; doi:10.1186/s12302-016-0080-y)
Supplement: Supplementary file 4 — 10.1186/s12302-016-0080-ySchematic overview of a diffusion cell. Skin samples are clamped in between the receptor and donor chamber and fixed by a metal clamp (for clarity, the metal clamp is not shown in this Figure); the receptor chamber is filled with the corresponding receptor medium which can be temperature-controlled via an outer chamber; test compounds are applied topically to the skin in the donor chamber and samples are drawn out of the receptor medium across the connection pipe of the receptor chamber; at the end of exposure remaining test substance may be washed off the skin surface and the diffusion cell may be dismantled; diffusion cell parts can be extracted separately for mass balance and all samples are analyzed. [file 12302_2016_80_MOESM4_ESM.pptx]

## Slide 1
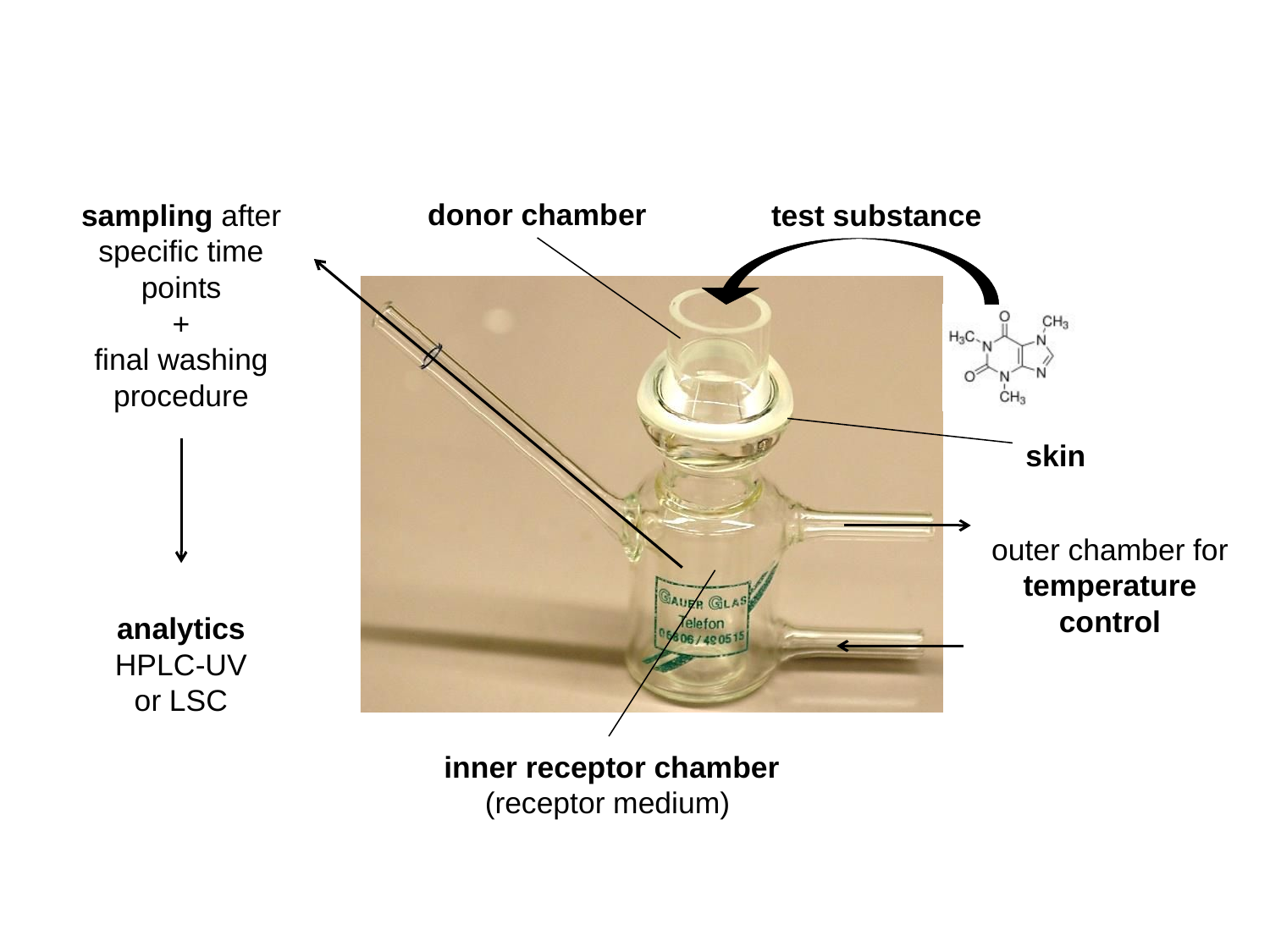

donor chamber
test substance
sampling after specific time points
+
final washing procedure
skin
outer chamber for temperature control
analytics
HPLC-UV
or LSC
inner receptor chamber (receptor medium)
